# Effect of exercise on multi-compartment lipid metabolism in murine models fed an obesogenic diet using non-targeted LC/MS

**DOI:** 10.3389/fnut.2025.1673002

**Published:** 2025-10-23

**Authors:** Rachana M. Gangadhara, Divyavani Gowda, Siddabasave Gowda B. Gowda, Gunjan Upadhyay, Nikhil Chainani, Vasundara Kain, Ganesh V. Halade, Shu-Ping Hui

**Affiliations:** ^1^Graduate School of Global Food Resources, Hokkaido University, Sapporo, Japan; ^2^Faculty of Health Sciences, Hokkaido University, Sapporo, Japan; ^3^Heart Institute, Division of Cardiovascular Sciences, Department of Internal Medicine, University of South Florida, Tampa, FL, United States; ^4^Morsani College of Medicine, University of South Florida, Tampa, FL, United States

**Keywords:** exercise, obesogenic diet, phospholipids, DHA, liquid chromatography, mass spectrometry

## Abstract

**Background:**

Chronic inflammation from obesogenic diets (OBD) disrupts lipid metabolism and contributes to non-communicable metabolic diseases. Exercise is a non-drug intervention that reduces inflammatory metabolic diseases by improving lipid metabolism. However, there are research gaps in understanding how the lipid metabolites are altered by exercise under an OBD.

**Methods:**

Two-month-old male C57BL/6 J mice were fed a control (CON diet-Standard Lab Chaw-3.4%w/w Teklad Global #2918) or OBD for 10 months, then assigned to sedentary (Sed) or exercise (Exe) groups for 2 weeks. This study aims to examine changes in lipid metabolites in plasma, tissues, and feces of mice using untargeted liquid chromatography/mass spectrometry (LC/MS). Echocardiography was used to assess the impact of OBD on heart function.

**Results:**

A total of 363 lipid molecular species were identified and characterized in the murine samples by retention time behavior and MS/MS spectral annotation. Multivariate analysis showed a distinct group separation between CON and OBD groups in both Sed and Exe groups. Phospholipids acylated with docosahexaenoic acid (DHA) are the key metabolites responsible for group separation in tissues and plasma, whereas in feces, glycerolipids, mainly monoacylglycerols. Lysophosphatidylethanolamine (LPE 22:5) was significantly upregulated in the liver, plasma, and left ventricle of the OBD mice in both Sed and Exe groups, contradictorily DHA containing phosphatidylglycerol [PG (22:6/22:6)] was significantly downregulated. Exercise modestly modulated the lipid profile under OBD, lowering plasma ceramides and partially reversing lipid alterations in feces. Interestingly, exercise combined with a control diet led to an increase in gut-microbiota-derived short-chain fatty acid esters of hydroxy fatty acids.

**Conclusion:**

Chronic OBD induces distinct lipid alterations across multiple biological compartments. Short-term exercise provides modest improvements, with stronger benefits when combined with a balanced diet.

## 1 Introduction

Acute physiological Inflammation is the biological response to harmful stimuli, such as pathogens or tissue injury, serving as a defense mechanism to promote healing and restore homeostasis (1). However, if this response persists over time, it leads to a prolonged presence of immune system cells, leading to chronic or unresolved inflammation (2). Chronic inflammation is known to play a key role in non-communicable diseases (NCDs), such as obesity, type 2 diabetes, cardiovascular disease, cancer, autoimmune, and neurodegenerative disorders (3). Evidence shows that lifestyle factors, such as inactivity, stress, lack of sleep (night shift), and diets rich in saturated fats, sugars, and ultra-processed foods, known as obesogenic diets (OBD), play a significant role in promoting inflammation (4). This inflammation contributes to increased fat accumulation, insulin resistance, and a chronic, low-grade inflammatory condition that raises the risk of metabolic disorders and organ damage over time (5). Physical exercise, recognized as a nonpharmacological therapy, is effective against the risk of chronic inflammatory diseases, including cardiometabolic disorders, cancer, and neurodegenerative diseases (6). Studies have shown that exercise can lower pro-inflammatory eicosanoids, cytokines such as TNF-*α* and IL-6, and activate anti-inflammatory pathways, often regardless of weight loss (7).

Studies have explored the detrimental impact of obesogenic inflammatory diets on organ-specific inflammation and metabolic dysfunction. Intake of an obesogenic diet regularly disrupts gut microbial diversity by shifting communities toward Firmicutes, Proteobacteria, and Tenericutes, and increasing the Firmicutes/Bacteroidetes ratio (8–10). This imbalance weakens epithelial tight junctions, leading to increased intestinal permeability. In obese and diabetic mouse models, such dietary patterns increase intestinal permeability, leading to metabolic endotoxemia and sustained low-grade inflammation (11). A murine study on an OBD demonstrated that it induced hepatic inflammation, as evidenced by elevated liver enzymes (ALT, AST) and proinflammatory markers (CCR2, TNF-*α*, and IL-1β), and disrupted the *ω*-6 and ω-3 fatty acid balance (12). A study on the effect of diet on cardiac health used quantitative proteomics to demonstrate that prolonged high-fat feeding changes the protein networks in the mouse heart. These changes affect oxidative stress, apoptosis, and inflammatory pathways, suggesting that diet-induced lipotoxicity contributes to early cardiovascular damage (13). Feng et al. showed that high-fat diets increase splenic TNF-*α* levels and boost TLR4 and NF-κB transcripts, while moderate treadmill exercise restores these to normal responses (14). Recent studies on mouse models fed OBD reveal that voluntary exercise helps restore resolution mediators like Docosahexaenoic acid (DHA) and Eicosapentaenoic acid (EPA) in the heart and spleen, decreases pro-inflammatory lipid mediators, and enhances immune regulation (7). Research consistently shows that an obesogenic diet is a key factor in systemic inflammation, disrupting metabolic and immune balance across multiple organs and contributing to chronic disease development (15, 16).

Lipids are biologically active molecules that function as energy sources, structural elements of membranes, and key players in signaling pathways related to inflammation and immune responses (17). Lipid-derived mediators from polyunsaturated fatty acids, such as prostaglandins, leukotrienes, and specialized resolving mediators (SPMs), are essential for initiating and resolving inflammation (18, 19). Disruptions in lipid metabolism can interfere with these signaling pathways and promote the development of inflammatory diseases (20). Therefore, studying the lipid profile is essential, as it offers valuable insights into the disease mechanisms. Thus, lipidomic profiling has become a valuable method for studying how lipids regulate inflammation and for discovering potential biomarkers and targets for therapy (21). This study investigated the impact of a two-week voluntary wheel running exercise on lipid alterations after long-term exposure to an OBD, which reflects dietary patterns rich in ultra-processed foods and is commonly linked to chronic inflammation. Using an untargeted lipidomics approach, we comprehensively profiled lipid alterations across plasma, feces, and multiple organs. This study design allowed us to capture systemic and tissue-specific lipid remodeling in response to both prolonged OBD feeding and subsequent exercise intervention. The significance of this study is that while long-term OBD consumption induces lipid imbalance in a tissue-dependent manner, a short, two-week period of low-intensity voluntary wheel running induces only modest changes in lipid composition. These findings suggest that brief volunteer slow-paced physical activity might be inadequate to reverse lipidomic and inflammatory changes caused by prolonged dietary stress.

## 2 Materials and methods

### 2.1 Animal care compliance

All animal experiments were conducted in strict accordance with the “Guide for the Care and Use of Laboratory Animals” (8th Edition, 2011) and the AVMA Guidelines for the Euthanasia of Animals (2013 Edition) The protocols were reviewed and approved (approval number 7371R) by the Institutional Animal Care and Use Committees at the University of South Florida, Tampa, Florida (7).

### 2.2 Study design and diet intervention

**Figure 1:**
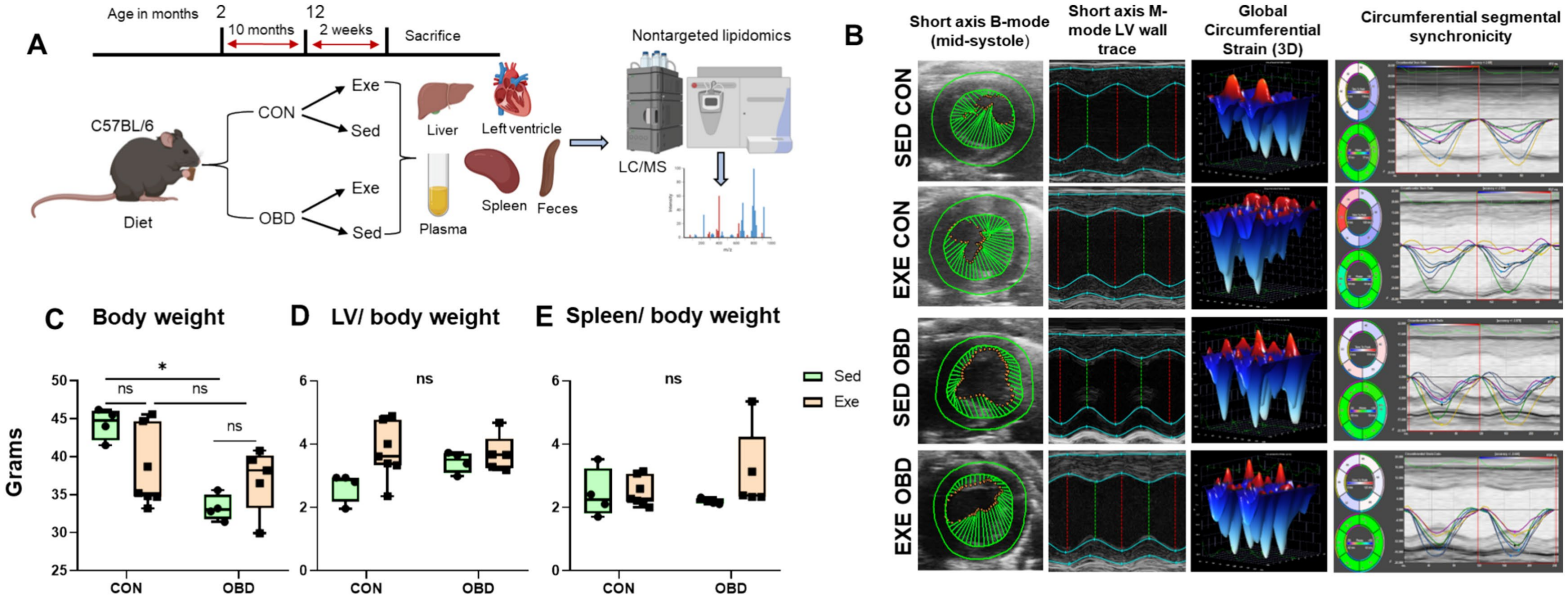
**(A)** Study design strategy for lipidomics analysis of diet and exercise relationship. **(B)** Representative left-ventricular (LV) short-axis B-mode and M-mode images with speckle tracking-based longitudinal three-dimensional (3D) myocardial strain and wall trace with segmental synchronicity images from control (CON) and obesogenic diet (OBD) mice groups in sedentary (Sed) and exercise (Exe) conditions. Box and Whisker plots depicting changes in **(C)** body weight (BW) in grams, **(D)** LV/BW ratio, and **(E)** Spleen/BW ratio in CON and OBD mice in Sed and Exe groups. Two-way ANOVA, Tukey’s multiple comparisons test was applied (*p < 0.05, ns, non-significant). Statistical comparisons were performed between Sed-CON vs. Exe CON, Sed OBD vs. Exe OBD, Sed CON vs. Sed OBD, and Exe CON vs. Exe OBD groups. Sed CON (*n* = 4), Sed OBD (*n* = 4), Exe CON (*n* = 7), and Exe OBD (*n* = 5) for LV and spleen.

Male C57BL/6 J mice (2 months old) were purchased from Jackson Laboratory and divided into two diet groups: control (CON-3.4% fat (10% safflower oil), Teklad #2918) and obesogenic (OBD-10% safflower oil, Research Diets #D11102001), providing 18 and 22 kcal, respectively. Detailed composition of the diet is given in Supplementary Table S1. Each group was further split into sedentary (Sed) and exercise (Exe) subgroups. The exercise groups underwent 2 weeks of voluntary wheel running. Mice were euthanized under anesthesia at ZT15 to minimize the biological variation linked to the circadian rhythm cycle (7). Then the liver, LV, spleen, feces, and plasma samples were collected and subjected to untargeted lipidomics as shown in Figure 1A.

### 2.3 Transthoracic speckle tracking echocardiography and necropsy

Echocardiography and necropsy were performed similarly to our previous study (7). In brief, echocardiography was conducted in a blinded manner using a Vevo 3,100 system with an MX400 probe under 1.5% isoflurane anesthesia at 37 °C, maintaining heart rates of 400–550 beats/min. Following echocardiography, mice were sacrificed at ZT = 15 under 3–5% isoflurane in oxygen following intraperitoneal heparin (4 IU/g) administration. Blood was collected from the carotid artery, and the LV (left ventricle), RV (right ventricle), lungs, and spleen were collected, weighed, and snap-frozen at −80 °C for biochemical and molecular analysis.

### 2.4 Chemicals and reagents

Mobile phase and extraction solvents of LC/MS grade, including methanol, chloroform, isopropanol, and ammonium acetate, were purchased from Wako Pure Chemical Industries, Ltd. in Osaka, Japan. Oleic acid-d9 and EquiSPLASH lipidomix were acquired from Avanti Polar Lipids located in Alabaster, AL, United States.

### 2.5 Lipid extraction

Total lipid extraction from samples, including the left ventricle, spleen, liver, feces, and plasma, was conducted using the Folch method with minor modifications as established earlier in our laboratory (22). Deep-frozen tissue samples were weighed, ice-cold methanol was added (100 μL /10 mg) and homogenized using a Bead Mill 4 (Fisherbrand) for two cycles of 30 s. Next, exactly 100 μL of the methanol homogenate was transferred into a 1.5 mL Eppendorf tube for further extractions. In the case of plasma samples, 50 μL of plasma was directly extracted by the addition of 100 μL of methanol. In both tissues and plasma,100 μL of the premixed EquiSPLASH lipidomix (1 μg/mL) and oleic acid-d9 (10 μg/mL) of internal standard in methanol was added and vortexed for 30 s at 3500 rpm at room temperature. Following this, 400 μL of chloroform and 100 μL of milli-Q water were added, then vortexed for 5 min at 3500 rpm. The mixture was centrifuged at 15,000 rpm for 10 min at 4 °C, and the organic layer was transferred to a new Eppendorf tube. The residue was re-extracted with an additional 400 μL of chloroform. The combined organic extracts were dried under a vacuum at 4 °C, redissolved in 100 μL of methanol, and transferred to LC vials. 10 μL of each sample was injected into the LC/MS.

### 2.6 Lipidomic analysis by HPLC/LTQ-Orbitrap MS

Untargeted lipidomics was conducted using high-performance liquid chromatography (HPLC) based on the LC-20 AD UFLC system (Shimadzu Corp., Kyoto, Japan) coupled with Orbitrap LTQ XL (Thermo-Fisher Scientific Inc., San Jose, CA) mass spectrometry. Lipid separation was achieved using an Atlantis T3 C18 column (2.1 mm × 150 mm, 3 μM, Waters, Milford, MA). The HPLC system consists of three mobile phase solvents: solvent A is 10 mM ammonium acetate, solvent B is isopropyl alcohol, and solvent C is methanol. The elution gradient follows the same protocol as our previous research for both negative and positive modes (22). Mass spectrometric analysis was conducted using an Orbitrap LTQ XL instrument (Thermo-Fisher Scientific Inc., San Jose) in both positive and negative ionization modes. The capillary temperature was set at 330 °C, employing nitrogen as the sheath and auxiliary gases at flow rates of 50 and 20 units, respectively. For the negative ion mode, the source voltage was established at 3 kV and the capillary voltage at 10 V, spanning an *m/z* range of 160–1900. In positive ion mode, these voltages were modified to 4 kV and 25 V, with an *m/z* scan range from 150 to 1950. High-resolution MS1 spectra were collected in Fourier transform mode at a resolution of 60,000. Additionally, low-resolution MS/MS spectra were recorded using ion trap mode with a collision energy of 40 V.

### 2.7 LC/MS data processing and lipid quantification

The raw data were processed using MS-DIAL software version 4.9 for alignment, peak extraction, and peak identification. Peak area integration was conducted with Xcalibur software version 4.0. To confirm lipid molecular species identification, MS and MS/MS spectra were utilized. The peak area ratios of annotated lipids to the internal standard were adjusted by multiplying them by the concentration of the added internal standard for relative quantification of lipid molecular species. Additionally, concentrations were normalized based on the weight of the samples used for lipid extraction.

### 2.8 Statistical analysis

Data were analyzed using Microsoft Excel 2019 and GraphPad Prism 8 software (San Diego, CA, United States), presenting the mean and standard deviation for Sed CON (*n* = 4), Sed OBD (*n* = 4), Exe CON (*n* = 7), and Exe OBD (*n* = 5) for liver, plasma, LV, and spleen. For feces, Sed CON (*n* = 15), Sed OBD (*n* = 13), Exe CON (*n* = 7), and Exe OBD (*n* = 5). Two-way ANOVA, multiple comparisons test was applied (*p < 0.0001, **p < 0.001, ***p < 0.01, ^#^*p* < 0.05, ns, non-significant). Statistical comparisons were performed between Sed CON vs. Exe CON, Sed OBD vs. Exe OBD, Sed CON vs. Sed OBD, and Exe CON vs. Exe OBD groups. Sparse Partial Least Squares Discriminant Analysis (sPLSDA), Volcano plot (*p* < 0.05) analysis, and cluster correlation analysis were performed using MetaboAnalyst version 6.0,^1^ with access on 9 July 2022.

## 3 Results

### 3.1 Impact of obesogenic diet and exercise on heart function and integrative multivariate lipid analysis

To evaluate the impact of OBD combined with the Exe on cardiac function, high-resolution echocardiography was performed. No significant difference was observed across experimental groups in cardiac strain, systolic function, as assessed by ejection fraction and ventricular synchrony (Figure 1B). Cardiac strain reflects myocardial deformation during systole and diastole, while ejection fraction quantifies the heart’s pumping efficiency. Synchrony refers to the coordinated contraction of different heart regions. The absence of significant changes in these parameters indicates that neither the OBD nor Exe adversely affected cardiac function. Necropsy analysis revealed a significant increase in body weight in Sed mice on the CON diet compared to those on the OBD, whereas no differences in body weight were observed under exercise conditions (Figure 1C). Additionally, the left ventricle-to-body weight ratio and spleen-to-body weight ratio remained unchanged across all groups (Figures 1D,E), indicating that the diet in combination with voluntary exercise does not have adverse systemic changes.

**Figure 2:**
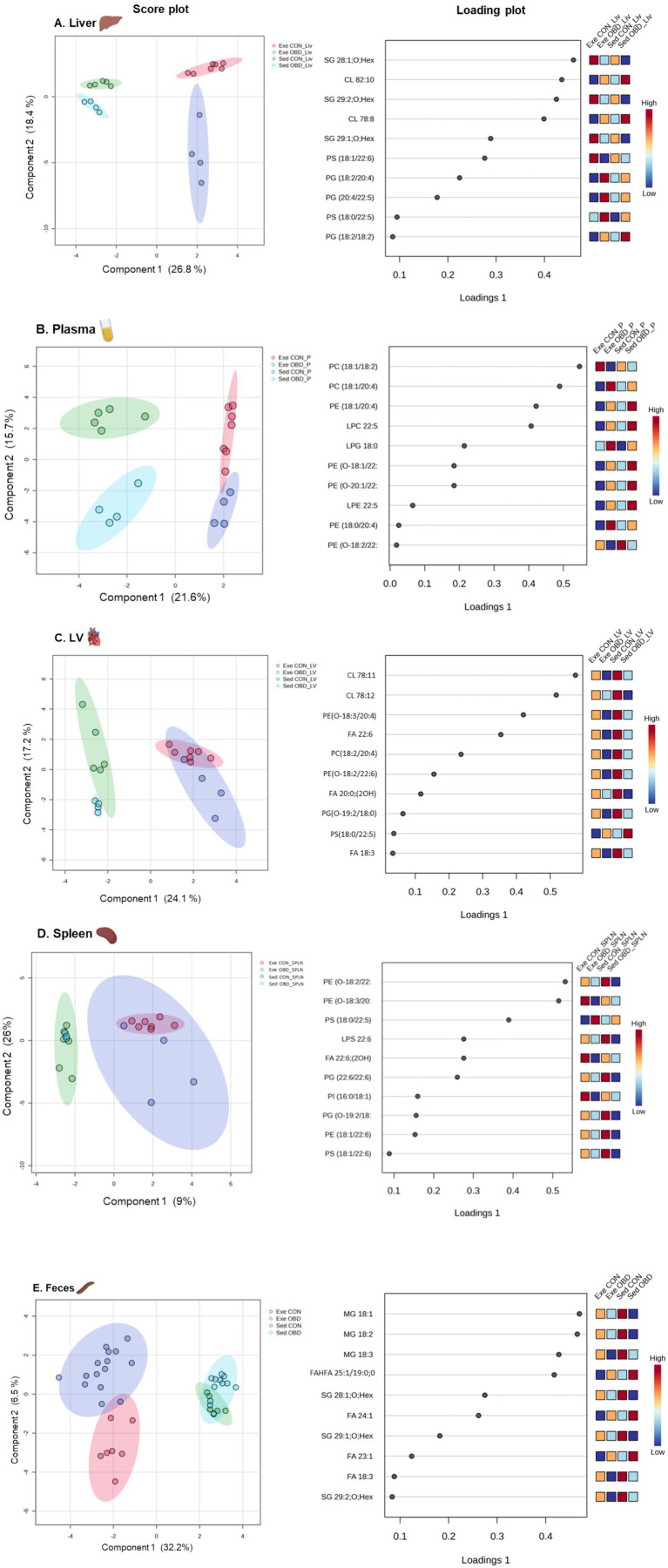
Multivariate analysis of lipid metabolites across five tissue types. Sparse partial least squares discriminant analysis (sPLSDA) score plot and variable importance in projection (VIP) loading plot illustrating the separation of groups and key contributing lipid species in **(A)** Liver, **(B)** Plasma, **(C)** Left Ventricle (LV), **(D)** Spleen, and **(E)** Feces. Sed CON (*n* = 4), Sed OBD (*n* = 4), Exe CON (*n* = 7), and Exe OBD (*n* = 5) for liver, plasma, LV, and spleen. For feces, Sed CON (*n* = 15), Sed OBD (*n* = 13), Exe CON (*n* = 7), and Exe OBD (*n* = 5).

This study shares an overlapping animal cohort with previous research that focused on targeted lipidomics in the heart and spleen related to exercise and aging (7). However, it differs in scope and biological focus, using a multi-organ, untargeted lipidomics approach to map systemic lipid mediator networks across the LV, spleen, liver, and plasma. Lipid analysis was performed with HPLC/LTQ-Orbitrap-MS in both negative and positive ionization modes. The list of all the identified lipid metabolites, along with the corresponding statistical significance values (*p*-values) for differences across groups in all the tissue samples (left ventricle, spleen, liver), plasma, and feces, was provided in the supporting information (Supplementary Table S2). A multivariate analysis was performed to assess the lipidomic changes by the obesogenic diet and exercise on multiple compartments, such as various organs (liver, spleen, LV), plasma, and feces. The results of the Sparse Partial Least Squares Discriminant Analysis (sPLSDA) score plot for all identified lipid species in the liver and plasma are shown in Figures 2A,B, respectively. The score plot demonstrates a clear separation among all groups, accounting for 45.2% of the total variance in the liver and 37.3% in plasma. The loading scores of the top 10 lipid species, such as sterols (SG) and glycerophospholipids (GPs), contributing to the group separation were shown in the loading plot. Among GPs, phosphatidylglycerol (PG) and phosphatidylserine (PS) contributed to group separation in the liver, whereas phosphatidylethanolamine (PE) and phosphatidylcholine (PC) contributed to group separation in plasma. Interestingly, fatty acid (FA) 22:5 acylated GPs were the largest contributors. The score plot analysis for the LV and spleen showed distinct group separation between the control and OBD, accounting for 41.3 and 35% of the total variance, respectively. However, no significant lipidomic changes were observed between the CON groups of Exe and Sed in the LV and spleen, which were similar in the OBD groups as well. The loading plot for the LV and spleen also revealed that GPs, such as PE, PC, PG, and PS, acylated with FA 22:6 primarily contributed to group separation (Figures 2C,D). Additionally, Cardiolipin (CL) 78:11 and CL 78:12 were the largest contributors in the LV. The sPLSDA score plot of feces revealed marked lipid alterations between the Sed and Exe groups, as illustrated in Figure 2E, accounting for 38.7% of the total variance. The loading plot revealed that monoacylglycerol (MG 18:1, 18:2, 18:3), stigmasterol hexoside (SG), and long-chain monounsaturated fatty acids are responsible for the separation of the groups.

### 3.2 Diet and exercise induced lipid variation in the liver and plasma

**Figure 3:**
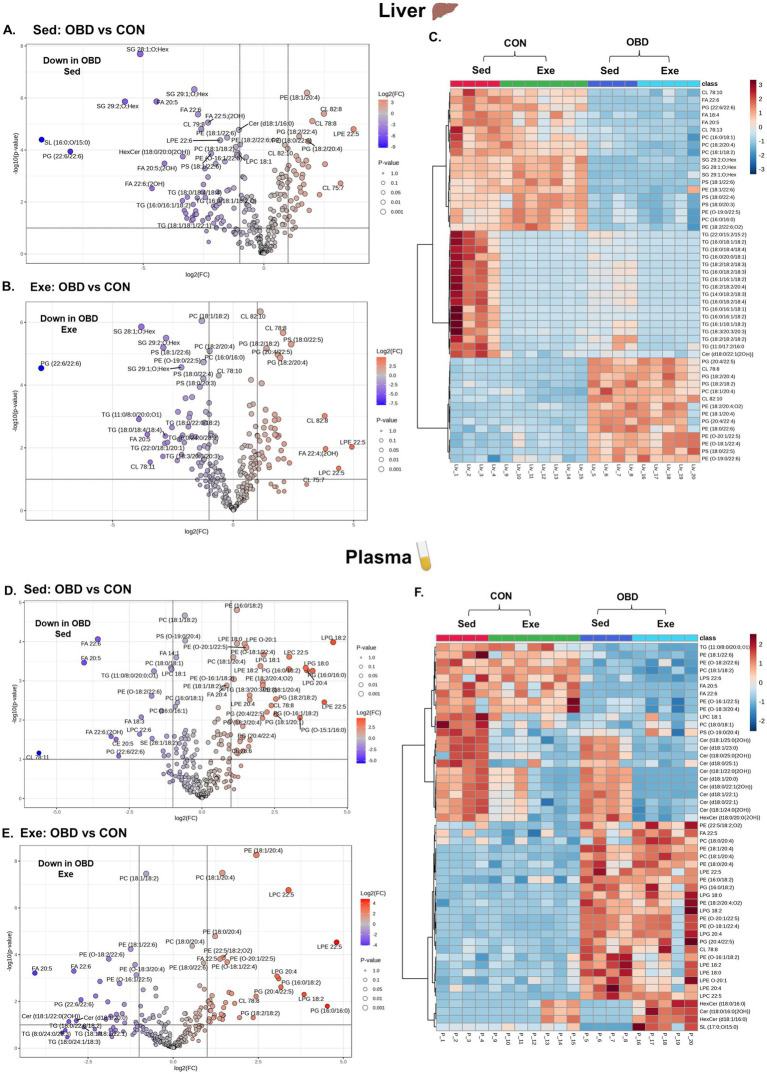
Volcanic plot representing significantly altered lipids (t-test, *p* < 0.05) in the liver **(A)** Exe OBD vs. CON, **(B)** Sed OBD vs. CON, and plasma **(D)** Exe OBD vs. CON, **(E)** Sed OBD vs. CON. Hierarchical correlation analysis of exercise and sedentary in **(C)** Liver and **(F)** plasma (clustering method: Ward, distance measure: Euclidean). Sed CON (*n* = 4), Sed OBD (*n* = 4), Exe CON (*n* = 7), and Exe OBD (*n* = 5) for liver and plasma.

Volcanic plots and the correlation analysis of Sed and Exe groups of mice between CON and OBD, showing lipid changes in the liver and plasma, are depicted in Figure 3. A volcanic plot illustrates the graph of -log 10(*p*-value) vs. log 2 (fold change), representing the significantly altered lipid molecular species. The volcano plot results indicates that in the liver most of the phospholipids such as PC, PS, containing DHA(FA 22:6), triacylglycerol (TGs) and SG lipids, including SG 28:1; O; Hex, SG 29:1; O; Hex, and SG 29:2; O; Hex are significantly downregulated (represented in blue) in the Sed OBD as shown in Figure 3A. These lipid changes remained largely unchanged in the Exe OBD group (Figure 3B), suggesting that exercise did not fully restore the lipid profile to that of the control. The heat map (Figure 3C) represents the top 50 altered lipids of the Sed and Exe groups. It was observed that TG lipid species are decreased in the Exe OBD groups compared to the Sed OBD group, indicating a possible exercise-mediated reduction in hepatic triglyceride accumulation. Although TG levels were higher in the Sed CON group, they were significantly lower in the Exe CON group, demonstrating that combining a control diet with exercise offers an even greater protective effect against liver triglyceride accumulation. Additionally, in the Sed OBD groups, there was a significant decrease in DHA acylated PE and PS lipids, while arachidonic acid (AA) acylated PC, PE, PG, and PS lipids increased compared to Exe OBD. These findings indicate a shift toward a pro-inflammatory lipid profile under the obesogenic diet, with voluntary slow-paced exercise showing no substantial effect in reversing these lipid alterations.

Plasma lipidomic analysis revealed a clear dietary influence, shown in volcanic plots in Figures 3D,E. Omega-3-derived free fatty acids, including FA 20:5 and FA 22:6, along with DHA-acylated glycerolipids, were significantly downregulated in both Sed and Exe OBD groups compared to controls. Conversely, glycerophospholipids containing AA (FA 20:4), such as PC (18:1/20:4), PE (18:1/20:4), and lyso-phosphatidylglycerol (LPG) 20:4, were significantly upregulated in Sed and Exe OBD groups, suggesting a shift toward a pro-inflammatory lipid profile under obesogenic diet stress. The heatmap (Figure 3F) further revealed that AA-containing phospholipids, such as PC, PE, and lyso-phosphatidylethanolamine (LPE), remained relatively unchanged in the Exe OBD group. Ceramide levels were increased in the plasma of Sed OBD mice but decreased with exercise, leading to lower ceramide levels in the Exe OBD group. This suggests that exercise alone can prevent OBD-induced ceramide buildup. Likewise, ceramide levels, which were higher in the Sed CON group, were reduced in the Exe CON group, highlighting the protective role of exercise across different dietary conditions. A key observation in the liver and plasma was the strong downregulation of PG (22.6/22:6) in Sed and Exe OBD groups, regardless of exercise, indicating the disruption of phospholipid metabolism. The volcano plots comparing Exe OBD and Sed OBD groups for liver and plasma are shown in Supplementary Figures S1A,B, respectively.

### 3.3 Lipid variation in the left ventricle and spleen

**Figure 4:**
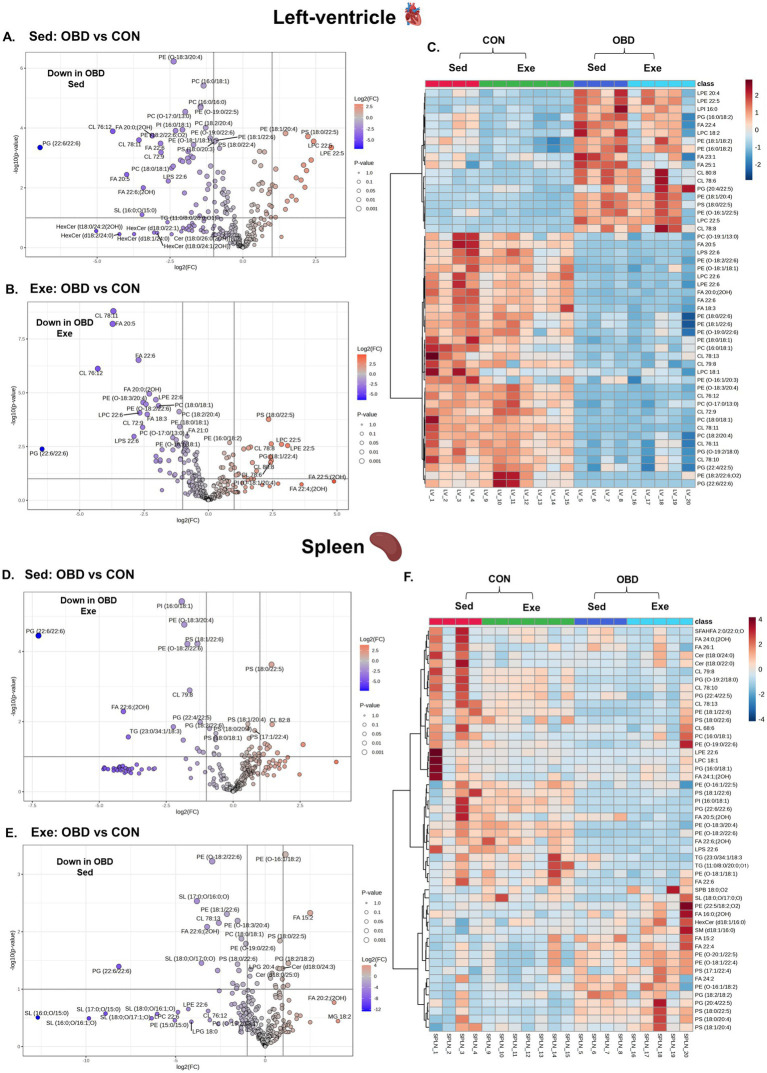
Volcanic plot representing significantly altered lipids (t-test, *p* < 0.05) in the left ventricle **(A)** Exe OBD vs. CON, **(B)** Sed OBD vs. CON, and spleen **(D)** Exe OBD vs. CON, **(E)** Sed OBD vs. CON. Hierarchical correlation analysis of exercise and sedentary in **(C)** Left ventricle (LV) and **(F)** Spleen (clustering method: Ward, distance measure: Euclidean). Sed CON (*n* = 4), Sed OBD (*n* = 4), Exe CON (*n* = 7), and Exe OBD (*n* = 5) for LV, and spleen.

Lipidomic profiling of the left ventricle (LV) and spleen showed significant changes in response to a chronic OBD, regardless of voluntary exercise, as illustrated in Figure 4. The volcano plot analysis (Figures 4A,B) revealed that several polyunsaturated fatty acids (PUFAs), especially EPA (FA 20:5) and DHA (FA 22:6), were significantly downregulated in the LV tissues of both Sed and Exe groups fed with OBD. Additionally, GPs containing DHA chains, such as PG (22:6/22:6), lyso-phosphatidylcholine (LPC) 22:6, and lyso-phosphatidylserine (LPS) 22:6, also showed significant reductions. These lipids are recognized for their anti-inflammatory and cardioprotective effects, implying a shift toward a more pro-inflammatory lipid profile in the OBD groups. Conversely, PS (18:0/22:5), LPC 22:5, and LPE 22:5 were significantly higher in the Sed and Exe OBD groups. This elevation may indicate membrane phospholipid remodeling in response to inflammation. The heatmap analysis (Figure 4C) showed that the 50 most affected lipids in the LV mainly belonged to the GP class. These findings support the volcano plot results, which also display reduced levels of GP lipids in the OBD group, suggesting that OBD leads to a depletion of anti-inflammatory phospholipids regardless of exercise.

Similar trends were observed in the spleen, where GP lipids acylated with DHA are decreased in the Sed and Exe mice of the OBD groups, as shown in Figures 3D,E. These changes indicate impaired inflammation resolution, considering the spleen’s role as a secondary lymphoid organ and immune cell reservoir (23). The volcano plots comparing the Exe OBD and Sed OBD groups for LV and spleen are shown in Supplementary Figure S1C,D, respectively. However, the overall alterations in spleen lipid levels were minor, evidenced by the slight variation visible in the heatmap (Figure 4F). This could be attributable to tissue-specific lipid metabolic processes. Additionally, there were no notable differences in lipid levels in the left ventricle and spleen between the Sed-CON and Exe-CON groups. This indicates that, under a control diet, dietary influences mainly determine tissue lipid composition, while short-term exercise exerts comparatively minimal impact on these tissues.

### 3.4 Impact of obesogenic diet and exercise on fecal lipids

**Figure 5:**
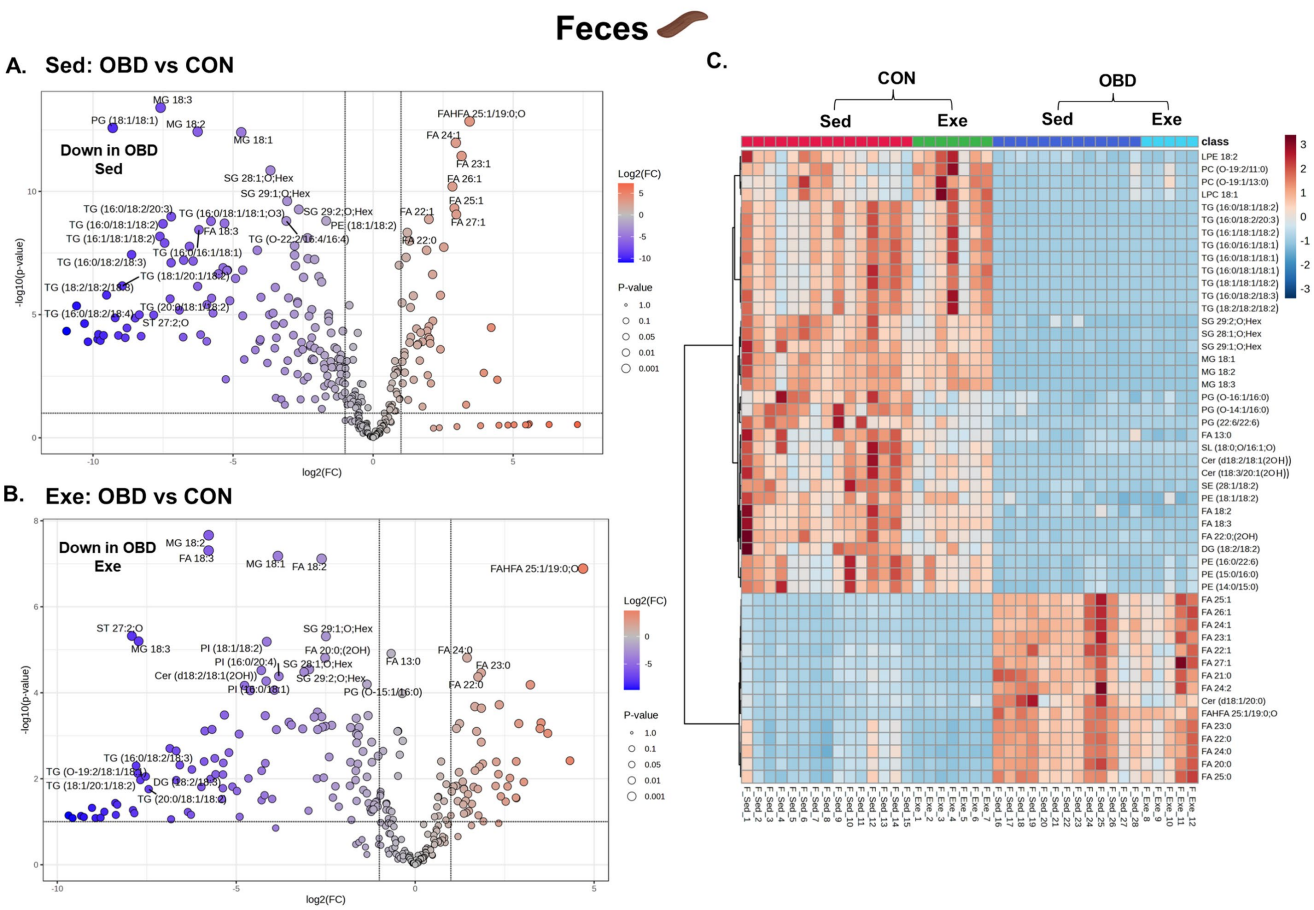
Volcanic plot representing significantly altered lipids (t-test, *p* < 0.05) in the Sed and Exe feces **(A)** Sed OBD vs. CON, **(B)** Exe OBD vs. CON. Hierarchical correlation analysis of **(C)** Exe and Sed groups (clustering method: Ward, distance measure: Euclidean). Sed CON (*n* = 15), Sed OBD (*n* = 13), Exe CON (*n* = 7), and Exe OBD (*n* = 5) for feces.

The comparative fecal lipidomic profiles of the Sed group and the Exe group are illustrated in Figure 5. In the Sed OBD group (Figure 5A), the volcano plot revealed a significant downregulation of TGs compared to the Exe OBD group, indicating reduced levels of fecal glycerolipids. In contrast, there was a notable upregulation of long-chain saturated and monounsaturated fatty acids in Sed OBD, particularly lipid species with carbon chain lengths > C20. In the exercise group, volcano plots (Figure 5B) demonstrated a consistent downregulation of glycerolipids (GLs), including TGs, MGs such as MG 18:1, 18:2, and 18:3, and SGs. These findings suggest that exercise under an obesogenic diet does not fully restore glycerolipid levels. The consistent decrease of GLs across Sed and Exe groups implies that the obesogenic diet could be the dominant factor driving lipid depletion, with exercise exerting some favorable influence. Supplementary Figure S1E displays the volcano plots comparing the Exe OBD and Sed OBD feces groups. Further insights were obtained from the heatmap-based correlation analysis of the top 50 altered fecal lipids (Figure 5C). This analysis revealed that GPs and ceramides were higher in the Sed and Exe CON group, indicating a more balanced and diverse fecal lipid profile under normal dietary conditions. In contrast, both Sed and Exe OBD groups showed elevated levels of long-chain saturated and monounsaturated fatty acids. These results suggest that although exercise slightly modulates the fecal lipid profile, its impact remains minimal under OBD conditions.

## 4 Discussion

**Figure 6:**
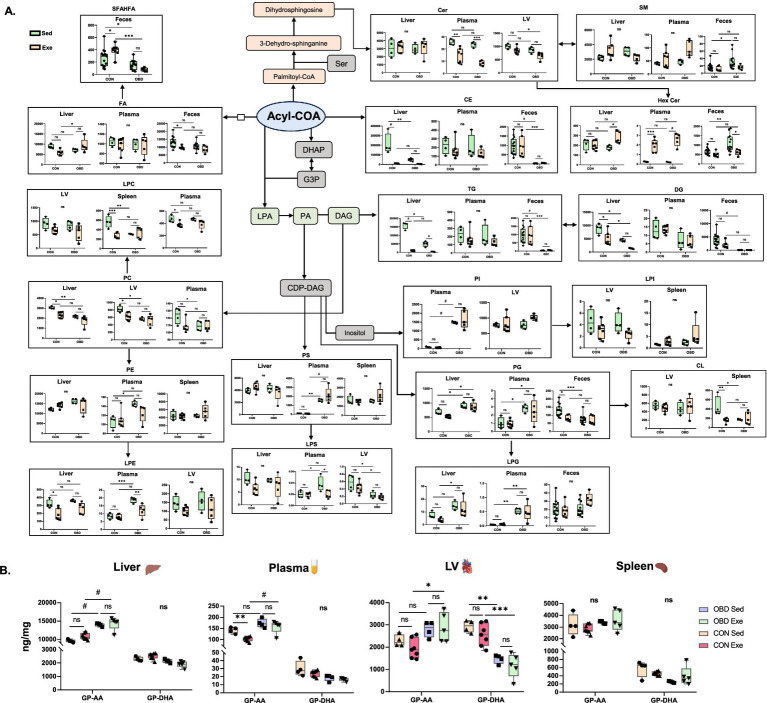
**(A)** Analysis of lipid biosynthesis pathways for the Sed CON, Sed OBD, Exe CON, and Exe OBD groups of liver, plasma, LV (left ventricle), spleen, and feces samples. The data are represented as mean ± SEM. The Y-axis represents the concentration of the lipid subclass in ng/mg. The X-axis represents the CON and OBD groups. **(B)** Concentrations of GPs containing AA and DHA. Two-way ANOVA, Sidak’s multiple comparisons test was applied (*p < 0.0332, **p < 0.0021, ***p < 0.0002, ^#^*p* < 0.0001, ns, non-significant). Statistical comparisons were performed between Sed CON vs. Exe CON, Sed OBD vs. Exe OBD, Sed CON vs. Sed OBD, and Exe CON vs. Exe OBD groups. Sed CON (*n* = 4), Sed OBD (*n* = 4), Exe CON (*n* = 7), and Exe OBD (*n* = 5) for liver, plasma, LV, and spleen. For feces, Sed CON (*n* = 15), Sed OBD (*n* = 13), Exe CON (*n* = 7), and Exe OBD (*n* = 5). The abbreviations are as follows: Glycerophospholipids (GPs), Arachidonic acid (AA), Decosahexanoic acid (DHA), Acetyl Coenzyme A (Acetyl CoA), Dihydroxyacetone Phosphate (DHAP), Glycerol-3-Phosphate (G3P). Lysophosphatidic Acid (LPA), Phosphatidic Acid (PA). Cytidine Diphosphate Diacylglycerol (CDP-DAG), Serine (Ser), Fatty acids (FAs), short-chain fatty acid esters of hydroxy fatty acids (SFAHFAs). Phosphatidylinositol (PI), Lyso-phosphatidylinositol (LPI), Phosphatidylethanolamine (PE), Lyso-phosphatidylethanolamine (LPE), Phosphatidylcholine (PC), Lyso-Phosphatidylcholine (LPC), Phosphatidylserine (PS), Lyso-Phosphatidylserine (LPS), Phosphatidylglycerol (PG), Lyso-Phosphatidylglycerol (LPG), Diacylglycerol (DAG), Triacylglycerol (TG), Cholesteryl Ester (CE), Ceramide (Cer), Hexosylceramide(HexCer), Sphingomyelin (SM), and Cardiolipin (CL).

The epidemic rise in obesity has led to multiple health issues, including cardiovascular diseases, diabetes, certain forms of cancer, and others (24). However, physical activity and exercise have been shown to have a positive impact on obesity and inflammation, leading to disease in both human and murine models (25). Exercise has been shown to trigger dynamic changes in lipids, affecting energy metabolism and inflammatory responses (26). Given this, our study assessed comprehensive lipid analysis in multiple compartments, such as various cardiometabolic organs (liver, heart, and spleen), plasma, and feces, to study the effect of exercise after chronic supplementation of OBD. Figure 6A and Supplementary Figure S1A illustrates a summarized view of this study through lipid biosynthesis pathway analysis based on KEGG pathways.^2^ The biosynthetic origins of the identified lipid classes have been reported in our previous study (27). An untargeted lipidomic study of tissues, plasma, and feces revealed the major lipidomic changes in the free fatty acids (FFA), fatty acyls (FA), glycerophospholipids (GPs), ceramides (Cer), and glycolipids (GLs) categories of lipids.

Polyunsaturated fatty acids (PUFAs), particularly omega (*ω*)-3 fatty acids, serve as precursors for various bioactive lipid mediators that influence inflammatory responses (26, 27). Typically, ω-3 PUFAs promote anti-inflammatory and resolving effects via specialized pro-resolving mediators (SPMs), whereas high consumption of ω-6 can result in pro-inflammatory conditions. Obesogenic diets are rich in ω-6 fatty acids, due to the wide use of seed-based vegetable oils and the consumption of processed foods (28). A recent study showed that long-term exposure to an OBD increases pro-inflammatory lipid mediators (DHA and EPA) in the heart and spleen. However, exercise reversed these effects by reducing macrophage-specific inflammatory genes, lipids, and affecting lipid metabolism genes in both organs (7). Our study also found a similar effect, although the results were not statistically significant, as shown in Supplementary Figure S1B. In contrast, we consistently observed the strong downregulation of phospholipids, such as PG and PE, containing 22:6 (DHA) fatty acids across all tissues and plasma, as depicted in Figure 6B. Phospholipids are a major class of lipids that serve as fundamental structural components of all biological membranes (29). Phospholipids containing PUFAs are crucial for maintaining membrane fluidity and regulating inflammatory responses. They are known to have anti-inflammatory and pro-resolving effects by producing specialized lipid mediators, such as lipoxins, resolvins, protectins, and maresins (30, 31). The significant decrease in PE and PG species with 22:6 fatty acids observed in our study suggests a possible disruption in the anti-inflammatory lipid reservoir during prolonged exposure to an obesogenic diet. Therefore, this suggests that short-term voluntary slow-paced exercise may have no significant effect on restoring phospholipid biosynthesis in OBD-fed mice.

Lysophospholipids originate from the phospholipids found in lipoproteins and cell membranes, modified by phospholipase enzymes (32). Research by Eisinger et al. found that PC, PI, and LPC are elevated in the serum of mice fed a high-fat diet (HFD), correlating strongly with glucose and cholesterol levels (33). The lipidomic study revealed a consistent reduction in circulating LPC species in liver and plasma associated with obesity and type 2 diabetes (34). LPC is produced when LDL undergoes oxidative modification through various mechanisms, and it has been proposed to possess both pro- and anti-atherogenic properties (32). However, a recent study by Martin et al., which investigated the effect of exercise on obesity by comparing it with sedentary and exercise groups of obese women, found a decrease in PC 40:4 levels and an increase in LPC 20:2 after the exercise intervention (35). Our study also found that PC and ether PE are downregulated in the Exe OBD compared to the Sed OBD in both plasma and liver. In contrast, they are downregulated in both the LV and spleen in both OBD groups. LPC 22:5 and LPE 22:5 (docosapentaenoic acid) species, which are also known to exhibit anti-inflammatory effects, significantly increase in the Exe OBD compared to the Sed OBD groups in liver, plasma, and LV. These results emphasize the role of glycerophospholipid remodeling in diet-related inflammatory conditions and reveal a reduction of protective lipid species across the tissues affected by metabolic stress. Our study found higher levels of long-chain saturated and monounsaturated fatty acids in the feces of the Sed OBD group. Research on morbidly obese people observed the accumulation of long-chain SFA, MUFAs, and PUFAs in their stool (36). This indicates a conserved disruption of gut lipid metabolism across different species, possibly related to impaired absorption or microbial-driven elongation, which may contribute to diet-induced effects on inflammation. Furthermore, we have identified a novel class of lipid SFHAFA (Short chain Fatty acid esters of hydroxy fatty acids) specific to the gut. A previous study showed a significant decrease in the colon content of mice fed a high-fat diet (37). Our study also showed a similar trend with SFAHFA, which significantly decreased in both Sed and Exe OBD groups of feces. However, SFHAFAs are significantly increased in the CON Exe compared to CON Sed (Figure 6B). This observation may suggest the effect of exercise on increasing SFHAFAs under controlled diet conditions.

Ceramides act as fundamental building blocks for more complex sphingolipids. Ceramide accumulation contributes to metabolic and cellular dysfunction, with recent studies highlighting its lipotoxic role in obesity, metabolic syndrome, type 2 diabetes, and cardiovascular conditions (38, 39). Another study reported that SM lipids with saturated acyl chains (C18:0, 20:0, 22:0, and 24:0) were increased in the serum of obese adults compared to the control group (40). On the other hand, studies have shown a decrease in ceramide and SM lipids in human plasma and serum following physical exercise intervention (35, 41). Boini et al. observed a significant decrease in the ceramides in the plasma of HFD (high-fat diet) mice compared to the control (42). In line with this, our study finds elevated plasma ceramide levels in the Sed OBD groups, but a significant decrease occurred after the exercise intervention. This suggests a visible effect of exercise on ceramide lipid metabolism and inflammatory improvements in obesogenic conditions. Glycerolipids play a vital role in energy storage (43). Dysregulation of glycerolipid metabolism can lead to lipid accumulation in non-adipose tissues, impair *β*-oxidation, promote lipotoxicity, and contribute to the development of metabolic diseases (44). The studies have demonstrated that Glycerolipids (TG, Diacylglycerol (DG), Cholesteryl Ester (CE)) are significantly increased in the liver, plasma, and heart of mice fed an HFD (45–47). A study by Jordy et al. comparing the obesogenic sedentary and exercise groups of mice found a decrease in TG, DG, and CE lipids in obese mice following exercise (48). Another study demonstrated that TG lipids were decreased in the plasma and liver of HFD-induced metabolic dysfunction-associated steatotic liver disease (MASLD) mice following exercise, indicating that exercise enhances lipid metabolism and slows the progression of MASLD (49). In line with these studies, our research also finds similar results, showing that TGs are decreased in the plasma of the Exe OBD group compared to the Sed OBD group. Earlier research indicates that obesity and an HFD lead to TG accumulation within the intestinal mucosa due to a reduced rate of intestinal lipid metabolism (50). In the present study, we observed a marked decrease in TG, MG, and DG levels in the feces of both Sed and Exe groups of OBD compared to the CON group. This suggests that altered intestinal lipid metabolism reduces the excretion of TG into the feces, consistent with the above study. In contrast, CON Exe mice exhibited increased feces TG levels, indicating enhanced lipid metabolism under standard diet and exercise intervention conditions.

The study reveals diverse lipid responses in mice under OBD across organs like the liver, plasma, LV, spleen, and feces. Lipidomic responses showed organ-specific and systemic patterns. DHA-containing phospholipids decreased in the liver, plasma, and LV, indicating reduced anti-inflammatory and cardioprotective lipids. AA–containing phospholipids increased in liver and plasma, suggesting a shift to a pro-inflammatory state. The spleen had minor changes, implying tissue-specific lipid regulation. Fecal lipids showed systemic effects, with glycerolipids decreasing and long-chain fatty acids increasing under OBD, with or without exercise. Overall, diet impacts lipid metabolism variably across organs, reflecting their functions. However, it also addresses several limitations: only one type of exercise (wheel running) was examined for a short duration (2 weeks). Whether different exercises, such as aerobic and high intensity with and without resistance, will exert a similar effect is unknown. The study included only male mice, excluding potential sex-based differences. The lipid concentrations reported in this study are semi-quantitative analyses, lack transcriptome data, and further targeted studies are required to determine their absolute levels and mechanistic role. Additionally, the relatively small number of samples in certain groups may limit the statistical power of our analysis. We focused on lipidomic changes; integrating transcriptomic, proteomic, or metabolomic data could improve understanding of the underlying biological processes. Additionally, our study used 10% w/w safflower oil; however, varying the percentage of saturated or polyunsaturated fats could lead to different results and direct measurements of inflammatory markers were not conducted in this study.

## 5 Conclusion

Our research investigated the effects of an obesogenic diet and exercise on tissue-specific lipid alterations and inflammation in the liver, left ventricle, spleen, feces, and plasma, utilizing HPLC/LTQ-Orbitrap-MS as the analytical method. The analysis showed that OBD disrupted lipid metabolism, marked by increased ceramides, decreased phospholipids containing DHA, and changes in glycerolipid and fatty acid compositions. Exercise intervention partially reversed these pro-inflammatory lipid signatures, primarily by reducing plasma ceramides and increasing anti-inflammatory LPC and LPE with FA 22:5 species. Feces lipidomics revealed the accumulation of very long-chain fatty acids, suggesting impaired absorption or microbial processing under OBD. These findings highlight the role of diet and exercise in regulating lipid-driven inflammation and modulating lipid balance. Our study suggests that combining exercise with a controlled diet is crucial for maintaining healthy metabolism and preventing cardiometabolic and non-communicable, chronic inflammation-driven disorders.

## Data Availability

The original contributions presented in the study are included in the article/Supplementary material, further inquiries can be directed to the corresponding authors.

## Footnotes

^1^ https://www.metaboanalyst.ca/

^2^ https://www.genome.jp/kegg/pathway.html
